# The anti-proliferative effects and mechanisms of low molecular weight scorpion BmK venom peptides on human hepatoma and cervical carcinoma cells *in vitro*

**DOI:** 10.3892/ol.2014.2336

**Published:** 2014-07-10

**Authors:** WEILING LI, YI XIN, YANG CHEN, XINLI LI, CUILI ZHANG, JING BAI, JIELI YUAN

**Affiliations:** 1 Department of Biotechnology, Dalian Medical University, Dalian, Liaoning 116044, P.R. China; 2 Department of Microecology, Dalian Medical University, Dalian, Liaoning 116044, P.R. China

**Keywords:** low molecular weight scorpion BmK venom peptides, anti-tumor effects, SMMC 7721, HeLa, apoptosis

## Abstract

Peptides from scorpion venom have been previously studied for use in the prevention and treatment of various types of cancer in folk medicine. The present study investigated the anti-proliferative effects and mechanisms of the low molecular weight (~3 kDa) BmK scorpion venom peptides (LMWSVP) on human hepatoma (SMMC 7721) and cervical carcinoma (HeLa) cells. The data indicated that LMWSVP inhibited the growth of SMMC 7721 cells, but had no effect on the growth of HeLa cells. SMMC 7721 cells were more sensitive, with a higher affinity, to LMWSVP as compared with HeLa cells. In addition, LMWSVP induced apoptosis of SMMC 7721 cells by upregulating the expression of caspase-3 and downregulating the expression of Bcl-2. These data provide an experimental basis for further purification and application of LMWSVP for use as an anti-tumor drug in clinical trials.

## Introduction

Cancer is the predominant cause of mortality in humans, and is a serious threat to human health. Many cancer patients, especially those in the metastasis stage, become resistant to traditional therapies (1). Therefore, identification of more efficient anticancer treatments, with lower toxicity, has been a prominent research topic. Besides traditional surgical intervention, chemotherapy and radiotherapy, attention should also be given to biological therapies in order to identify novel drugs from natural resourses.

Scorpion venom is a biological toxin that predominantly consists of neurotoxins, salts, peptides and enzymes with various biological functions (2). It has been reported that due to the low molecular weight of scorpion venom peptides, powerful effects can be exerted on excitable cells (3). A previous study reported cancer preventive and therapeutic effects of scorpion venom peptides in different animal models and cell culture systems, including cancers of the colon, prostate, and breast, as well as melanoma, glioma, and leukemia (4).

The Asian scorpion, *Buthus matensii Karsch,* (BmK) is widely distributed in Korea, Mongolia and China where it has been used as a Chinese medicine to relieve pain for thousands of years (5). The scorpion venom of BmK has not been shown to cause drug dependence, suggesting it could be a used as a potential pain-relief drug without risk of addiction (6). Although the biological activities of BmK venom have been extensively studied, the anti-tumor effects of BmK venom have been rarely investigated, with the exception of human glioma and leukemia (7,8).

The present study investigated whether the low molecular weight (~3 kDa) scorpion BmK venom peptides (LMWSVP) had anti-tumor effects, and determined the efficiency in human hepatoma (SMMC 7721) and cervical carcinoma HeLa cells. In addition, the anti-tumor mechanisms of LMWSVP on SMMC 7721 cells were investigated. These data provide an experimental basis for further purification and application of BmK scorpion venom as an anti-tumor drug in clinical trials.

## Materials and methods

### Preparation of the LMWSVP

Crude scorpion BmK venom powder was dissolved in distilled water and boiled for 1 h. The solution was centrifuged at 9,576 × g for 10 min. The supernatant was transferred to an Amicon Ultra tube (with molecular-weight cut-off of 5 kDa) and was centrifuged at 12,000 rpm for 15 min. The supernatant was then lyophilized for 16 h until it became a dry powder. The purified LMWSVP were weighed and dissolved in RPMI-1640 medium and stored at −20°C for further use.

### SDS-PAGE

The purified LMWSVP were subjected to SDS-PAGE. Briefly, 30 μl of LMWSVP was separated on a 15% SDS-PAGE gel for 2 h. The gel was stained by coommassie brilliant blue R250 overnight and then washed with 50% methanol and 7% acetic acid solution. The separated bands were visualized using a Geldoc systems (Bio-Rad, Hercules, CA, USA).

### Cell culture and treatment

The SMMC 7721 human hepatoma and human cervical carcinoma HeLa cells were purchased from the American Type Culture Collection (Manassas, VA, USA) and cultured in medium RPMI-1640 (Gibco-BRL, Carlsbad, CA, USA) supplemented with 10% fetus bovine serum (FBS) (Gibco-BRL) at 37°C with 5% CO_2_. When the cells were 80% confluent, they were harvested by 0.25% trypsin digestion (Gibco-BRL, USA). The cells were then treated with serial concentrations of LMWSVP and untreated cells were used as controls.

### Cell viability assay

The anti-proliferative effects of LMWSVP on SMMC 7721 and HeLa cells was examined by MTT assay (Sigma-Aldrich, St. Louis, MO, USA). Cells (5×10^5^) were seeded in 96-well plates and incubated for 24 h. LMWSVP (0.28, 0.70, 1.40, 2.80 and 5.60 μg/ml) was subsequently added to each well. The negative control group was treated with RPMI-1640 without LMWSVP. Each concentration of LMWSVP was repeated in five wells. After 24 h LMWSVP treatment, 20 μl of 5 mg/ml MTT solution (Amerco, USA) was added into each well and incubated for 4 h. Subsequently, 100 μl dimethyl sulfoxide was added to each well and the plates were assayed at a wavelength of 490 nm, using a Multiskan Ascent plate reader (Thermo Fisher Scientific, Waltham, MA, USA).

### Immunofluorescence labeling

SMMC 7721 and HeLa cells were adjusted to 3×10^6^ cells/ml and seeded in 6-well culture plates. Prior to the cells being added, sterile cover slips were placed into the 6-well plates. Cell climbing slices were generated until the cells had completely adhered to the cover slips. LMWSVP labeled with fluorescein isothiocyanate (C_21_H_11_O_2_N_5_; Sigma-Aldrich) was added to the cover slips and cultured for 8 h. The slices were washed with phosphate-buffered saline (PBS) prior to fixation with 4°C cold acetone for 10 min. The slices were dried overnight at room temperature in the dark, and then observed under a fluorescence microscope (DMI4000B; Leica, Mannheim, Germany).

### Western blot analysis

SMMC 7721 cells treated with 2.8 μg/ml of LMWSVP for 4 h were collected by centrifugation at 1,064 × g for 5 min at 4°C. The cells were then lysed in RIPA buffer, and 50 μg of total protein was separated by 10% SDS-PAGE for 2 h. The separated proteins were transferred to a nitrocellulose membrane (Pall Corporation, Port Washington, NY, USA) using a semi-dry blotter (Bio-Rad) for 1 h and then blocked with 5% non-fat milk for 1 h. The specific primary antibodies, at an optimized dilution, were incubated with the membrane [mouse anti-human Caspase-3 polyclonal antibody 1:1,000; rabbit anti-human Bcl-2 polyclonal antibody 1:1,000; mouse anti-human β-actin polyclonal antibody 1:10,000 (Santa Cruz Biotechnology, Inc., Santa Cruz, CA, USA)] and incubated overnight at 4°C. Following incubation with secondary horseradish peroxidase-conjugated antibody (goat anti-mouse and goat anti-rabbit,1:10,000) for 1 h, the protein bands were visualized by enhanced chemiluminescence (Santa Cruz Biotechnology, Inc.).

### Statistical analysis

SPSS 11.5 software (SPSS, Inc., Chicago, IL, USA) was used for the statistical analysis of data. One-way analysis of variance was used to examine the statistical significance between the groups. P<0.05 was considered to indicate a statistically significant difference.

## Results

### Molecular weight of LMWSVP

**Figure 1:**
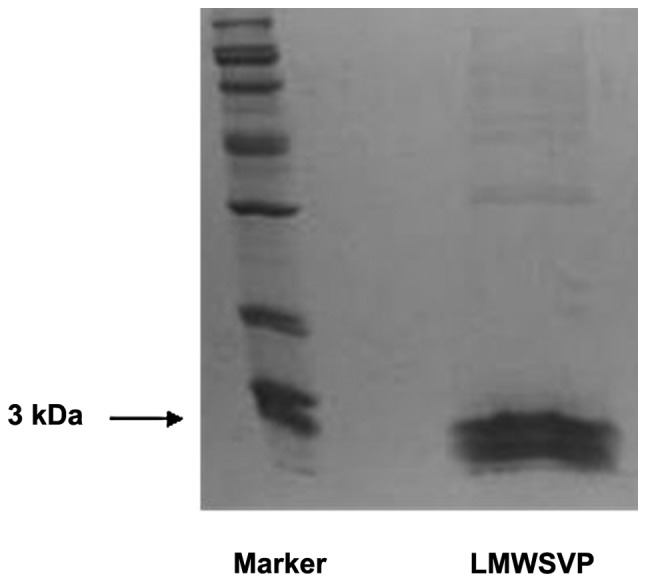
Molecular weight of the purified LMWSVP. The purified LMWSVP was subjected to SDS-PAGE. Based on the molecular weight marker, the molecular weight of LMWSVP was ~3 kDa. LMWSVP, low molecular weight BmK scorpion venom peptide; kDa, kilodaltons.

LMWSVP were subjected to SDS-PAGE to determine the molecular weight. The data showed that the molecular weight of LMWSVP was ~3 kDa (Fig. 1).

### LMWSVP inhibits cell proliferation of SMMC 7721 cells

**Figure 2:**
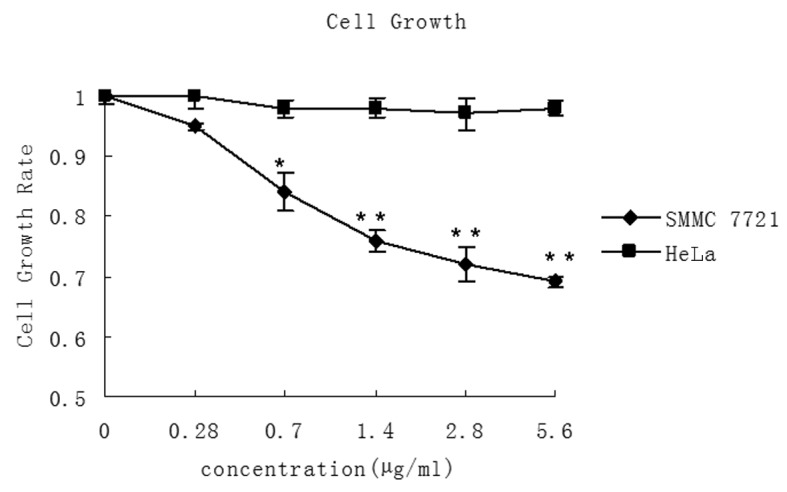
Effects of different concentrations of LMWSVP on the growth of SMMC 7721 and cervical carcinoma HeLa cells. Cells were treated with LMWSVP (0.28, 0.7, 1.4, 2.8 and 5.6 μg/ml) for 24 h. The negative control group was treated with RPMI 1640 without LMWSVP. The inhibiting rate of the cell growth was determined by MTT assay and expressed relative to that of non-treated group. The experiment was repeated in triplicate and a data are presented as the mean ± standard deviation. One-way analysis of variance was used to examine the statistical significance between the groups. ^*^P<0.05; and ^**^P<0.01 vs. HeLa cells. SMMC 7721, human hepatoma cells; LMWSVP, low molecular weight BmK scorpion venom peptide.

MTT assay was used to determine whether LMWSVP treatment inhibited the growth of SMMC 7721 and HeLa cells. It was observed that LMWSVP (0.28–5.60 μg/ml) treatment resulted in a dose-dependent decrease in SMMC 7721 cell growth. However, no significant difference was observed in the growth rate of HeLa cells (Fig. 2).

### Comparison of the affinity of SMMC 7721 and HeLa cells, to LMWSVP treatment

**Figure 3:**
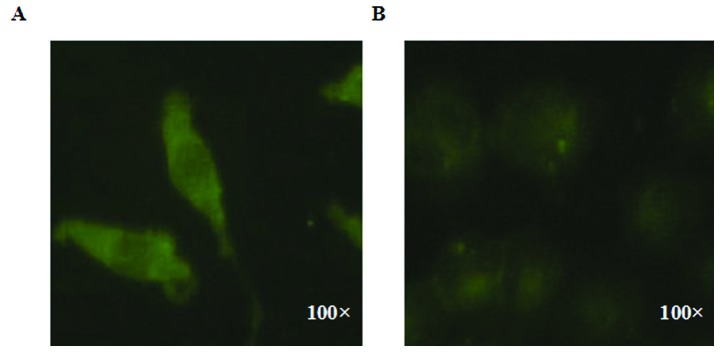
Fluorescein-labeled low molecular weight BmK scorpion venom peptide (LMWSVP) on SMMC 7721 human hepatoma and human cervical carcinoma HeLa cells. (A) SMMC 7721 and (B) HeLa cells were treated with fluorescein-labeled LMWSVP for 8 h and observed under a fluorescence microscope. The fluorescence of the SMMC 7721 cells with LMWSVP was stronger than that of the HeLa cells. Magnification, ×100.

SMMC 7721 and HeLa cells were exposed to immunofluorescent-labeled LMWSVP for 8 h. The results indicated that the fluorescence intensity of the labeled SMMC 7721 cells was higher than that of the labeled HeLa cells, when treated under the same same conditions of LMWSVP (Fig. 3). These data suggested that SMMC 7721 cells has a higher affinity for LMWSVP as compared with HeLa cells.

### Apoptosis-related protein expression

**Figure 4:**
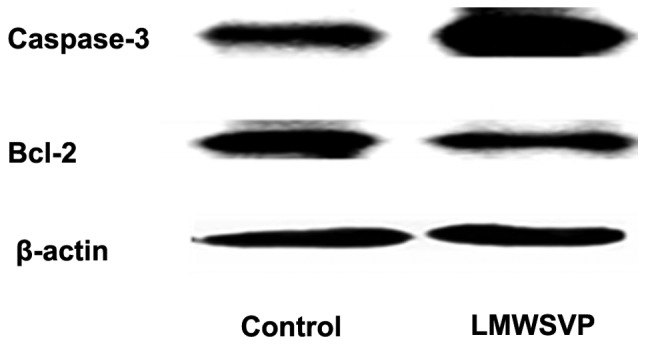
Effects of LMWSVP on apoptotic proteins. SMMC 7721 cells were treated with 2.8 μg/ml of LMWSVP for 4 h. Protein from the total cell lysate was subjected to western blotting for Caspase-3 and Bcl-2 protein expression. β-actin was used as an internal control. Representative results are shown from three independent experiments. LMWSVP, low molecular weight BmK scorpion venom peptide.

To investigate the anti-tumor mechanisms of LMWSVP, apoptosis-related protein expression (caspase-3 and Bcl-2) in SMMC 7721 cells exposed to LMWSVP was investigated. SMMC 7721 cells were treated with 2.8 μg/ml LMWSVP for 4 h prior to analysis by western blotting. The data showed that the expression of the pro-apoptotic protein, caspase-3, increased, whereas the expression of the anti-apoptotic protein Bcl-2 decreased, as compared with the non-treated control group (Fig. 4).

## Discussion

Scorpion venoms and toxins have been used as therapeutic drugs for cancer patients in traditional folk medicine practices (9). Scorpion venoms are a complex mixture of molecules, of which the majority are peptides exhibiting various functions (4). The present study has investigated the anti-tumor effects of LMWSVP on a human hepatoma and cervical carcinoma cell line.

The data of the present study have demonstrated that LMWSVP was able to induce growth inhibition in SMMC 7721 cells, but had no effect on the growth rate of HeLa cells. These data indicated that SMMC 7721 cells were more sensitive to LMWSVP as compared with HeLa cells. Additionally, results obtained from the immunofluorescent analysis revealed that immunofluorescently labeled LMWSVP had a higher affinity in SMMC 7721 cells as compared with HeLa cells. These may suggest a difference in the efficiency of LMWSVP between SMMC 7721 and HeLa cells.

The anti-tumor mechanisms of LMWSVP on the sensitive SMMC 7721 cell line was also examined. Since both extrinsic and intrinsic apoptotic pathways have been recognized as the predominant mechanisms of cell death in the majority of cellular systems (10), the apoptosis-related proteins were examined in SMMC 7721 cells treated with LMWSVP. Caspase-3 is an important enzyme of the intrinsic apoptotic pathway. Activation of caspase-3 is an indicator of activation of the intrinsic apoptotic pathway (11). In addition, Bcl-2 protein can protect cells from apoptosis by preventing activation of the caspase-3 dependent pathway (12). The results of the present study revealed that LMWSVP increased caspase-3 and decreased Bcl-2 protein expression, in SMMC 7721 cells. It could therefore be considered that the growth inhibition of SMMC 7721 cells by LMWSVP may be achieved through the induction of apoptosis.

The development of anti-tumor drugs from natural resources is an active line of investigation. Findings of the present study have shown that LMWSVP can prevent human hepatoma cell growth through an induction of apoptosis. As a biological toxin, the LMWSVP may be worth further investigation to determine its suitability as a novel anti-tumor drug in the future.

## Abbreviations

LMWSVP: low molecular weight BmK scorpion venom peptides
BmK: buthus matensii Karsch
FBS: fetal bovine serum
MTT: 3-(4,5-dimethylthiazol-2-yl)-2,5-diphenyltetrazolium bromide
SDS-PAGE: sodium dodecyl sulfate polyacrylamide gel electrophoresis
NC: nitrocellulose membrane
